# Curcumin Alleviates Palmitic Acid-Induced LOX-1 Upregulation by Suppressing Endoplasmic Reticulum Stress in HUVECs

**DOI:** 10.1155/2021/9983725

**Published:** 2021-08-22

**Authors:** Ruixi Luo, Lifeng Zhao, Shuaishuai Li, Peng Chen, La Wang, Honghong Yu, Kun Cai, Qi Yu, Weiyi Tian

**Affiliations:** ^1^Department of Immunology and Microbiology, School of Basic Medical Sciences, Guizhou University of Traditional Chinese Medicine, Guiyang, China; ^2^Stem Cell Therapy Research Center, Guizhou University of Traditional Chinese Medicine, Guiyang, China; ^3^Clinical Basis of Traditional Chinese Medicine Teaching and Research Section, School of Basic Medical Sciences, Guizhou University of Traditional Chinese Medicine, Guiyang, China; ^4^Department of Experimental Center, Guizhou University of Traditional Chinese Medicine, Guiyang, China

## Abstract

Excessive free fatty acid- (FFA-) induced endothelial lipotoxicity is involved in the pathogenesis of atherosclerosis. Endoplasmic reticulum (ER) stress is mechanistically related to endothelial lipotoxicity. Lectin-like oxidized low-density lipoprotein receptor-1 (LOX-1) is the major oxidatively modified low-density lipoprotein (OxLDL) receptor in endothelial cells and is highly abundant in atherosclerotic lesions. Curcumin reduces the LOX-1 expression; however, the mechanism underlying this effect remains unknown. In the current study, we explored whether curcumin ameliorates palmitic acid- (PA-) induced endothelial lipotoxicity and LOX-1 upregulation by reducing ER stress in human umbilical vein endothelial cells (HUVECs). We built endothelial lipotoxicity in vitro and found that LOX-1 was upregulated after PA stimulation, during which ER stress played an important role. Next, we observed that curcumin substantially alleviated PA-induced lipotoxicity by restoring cell viability, increasing angiogenesis, and decreasing lipid deposition. Furthermore, LOX-1 upregulation in HUVECs was blocked by curcumin, possibly via ER stress suppression. Overall, our findings demonstrated that curcumin alleviates endothelial lipotoxicity and LOX-1 upregulation, and ER stress inhibition may play a critical role in this effect.

## 1. Introduction

Current hypotheses suggest that high levels of free fatty acids (FFAs) are a risk factor for the pathology of atherosclerosis (AS) [1]. Endothelial cell dysfunction, which is the innermost layer of blood vessels, is considered an early indicator of AS [2]. In a previous study, we showed that palmitic acid (PA) induces lipotoxicity in endothelial cells [3], which contributes to the early phase of AS pathogenesis. However, the roles and detailed mechanisms of FFAs in the pathogenesis of AS remain unclear. LOX-1 was first identified in endothelial cells as the major OxLDL receptor and is highly abundant in atherosclerotic lesions [4]. Excessive binding of Ox-LDL to LOX-1 induces endothelial dysfunction, thus initiating atherosclerosis [5, 6]. PA is a stimulus that activates the LOX-1 expression and induces LOX-1 upregulation in macrophages [7, 8]. Therefore, preventing PA-induced lipotoxicity and LOX-1 upregulation in endothelial cells will contribute to avoiding endothelial damage.

The endoplasmic reticulum (ER) plays a key role in the cellular stress response and is considered a center for lipid and protein synthesis, folding, and assembly. We previously reported that PA induces lipotoxicity via the ER stress pathway in several types of cells, including pancreatic *β* cells [9], hepatocytes [10], and endothelial cells [3]. Furthermore, additional evidence has shown that ER stress is an initiating factor of lipotoxicity and atherosclerosis [11, 12]. Furthermore, ER stress is linked to a PA-induced increase in the LOX-1 expression in macrophages [13]. However, whether PA upregulates the LOX-1 expression via the ER stress signaling pathway in endothelial cells remains unknown.

*Curcuma longa* is a perennial herb widely cultivated in various regions of Asia. As a traditional Chinese herbal medicine, *Curcuma longa* has been applied for a long time in many traditional medicine systems to treat several cardiovascular diseases because of its capacity to “invigorate blood” [14]. Curcumin is the chief component of *Curcuma longa* and has various beneficial effects, including anti-inflammatory [15], antitumor [16], antioxidative [17], wound healing [18], and anti-infective effects [19]. Currently, curcumin is regarded as a health care product and is contained in the British Pharmacopoeia, United States Pharmacopeia, and European Pharmacopoeia [20]. Curcumin shows protective effects against AS in in vivo and in vitro models [21–23]. Guan et al. found that curcumin attenuates PA-induced apoptosis by alleviating ER stress in cardiomyocytes [24]. However, whether alleviating ER stress is essential for the effects of curcumin on endothelial damage and whether a link exists between curcumin and LOX-1 in PA-induced endothelial lipotoxicity remain to be elucidated. Hence, in this study, we examined whether curcumin ameliorates the PA-induced LOX-1 expression in primary endothelial cells and how ER stress is modulated by curcumin.

## 2. Materials and Methods

### 2.1. Preparation of Fatty Acids

First, 0.0641 g of PA (Aladdin, Shanghai, China) was dissolved in 2.5 ml of 100% ethanol to reach a final concentration of 100 mM. The solution was then mixed with 22.5 ml of 20% fatty acid-free bovine serum albumin (BSA; Solarbio, Beijing, China) in phosphate-buffered saline (PBS) at 50°C for 1 h, yielding a final stock solution of 10 mM. A solvent control (18% BSA) was prepared by mixing 2.22 ml of 100% ethanol with 20 ml of 20% fatty acid-free BSA. All stock solutions were stored at -20°C.

### 2.2. Cell Culture and Treatment

Human umbilical vein endothelial cells (HUVECs) were purchased from Zhongqiao Xinzhou Co., Ltd. (Shanghai, China). HUVECs were cultured in ECM (ScienCell Corporation, Billerica, USA) supplemented with 10% fetal bovine serum (FBS) and antibiotics (100 U/ml penicillin and 0.1 mg/ml streptomycin) at 37°C in 5% CO_2_. In this study, the control group refers to cells treated with BSA (100 *μ*M). For stimulation experiments, HUVECs were treated with PA or the chemical ER stressor tunicamycin (TM) (5045700001; Sigma, USA) for 24 h with or without pretreatment with curcumin (S1848; Selleck, Houston, USA) or the ER stress inhibitor sodium tauroursodeoxycholate (TUDCA) (abs816166; Absin, Shanghai, China) for 1 h.

### 2.3. Cell Viability

Cell viability was measured using a cell counting kit (CCK-8; Dojindo, Japan) after treatment. Briefly, the CCK-8 stock solution was diluted ten times with ECM, and 100 *μ*L was added to the plates. After 2–4 h, the absorbance at 450 nm was measured using a thermomicroplate reader (Thermo Instruments, USA).

### 2.4. Tube Formation Assay

The tube formation assay was assessed using Matrigel Basement Membrane Matrix (BD Biosciences, USA) [3]. The average number of tubules and dendritic length was calculated using ImageJ Pro Plus software.

### 2.5. Oil Red O Staining

Lipid accumulation in HUVECs was detected using Oil Red O staining. After rinsing twice with PBS, the stained cells were dissolved in isopropanol, and the absorbance was measured at 500 nm using a thermomicroplate reader.

### 2.6. Transmission Electron Microscopy (TEM)

After HUVECs were fixed, dehydrated, and embedded, the cells were stained with 5% uranyl acetate and lead citrate solution and then analyzed by TEM.

### 2.7. RNA Isolation and Quantitative Real-Time PCR

Total RNA from HUVECs was isolated using TRIzol Reagent (Ambion, Texas, USA), and then the RNA (1 *μ*g) was reverse transcribed into cDNA using RevertAid™ Master Mix (M1631; Thermo Scientific, Waltham, MA, USA) according to the manufacturer's protocol. Quantitative PCR (qPCR) was performed using SYBR Green PCR Mix (Bimake, Texas, USA) in a real-time PCR detector (Bio-Rad, CA, USA). The primer sequences used are listed in Supplemental Table S1, and the commercial *β*-actin primer was obtained from Sango Biotechnology (Shanghai, China). Each sample was tested in triplicate, and *β*-actin was used as an internal control.

### 2.8. Western Blotting

After total protein was extracted from HUVECs using RIPA lysis buffer (Solarbio, China), the protein concentrations were measured using an Omni-Easy™ BCA Protein Assay Kit (ZJ102L; EpiZyme, Shanghai, China). Protein extracts were separated by SDS-PAGE, transferred to a 0.22-*μ*m PVDF membrane, and incubated with primary antibodies against CHOP (A5462; Bimake), Bip (11587-1-AP; Proteintech, Wuhan, China), and LOX-1 (ab214427; Abcam, Cambridge, UK).

### 2.9. Immunofluorescence

Immunofluorescence staining was conducted as described in a previous study [3]. HUVECs were incubated using an anti-Bip antibody (Proteintech) and an anti-LOX1 antibody (10585-T26; Sino Biological). After staining with secondary antibodies (ab150077; Alexa Fluor® 488; Abcam, 1: 500) and DAPI, the cells were observed using a fluorescence microscope (Olympus, Japan).

### 2.10. Statistical Analysis

The data are presented as means ± SD for statistical analysis. The data were analyzed by one-way analysis of variance using either Duncan's multiple-range test or Student's *t*-test. A value of *P* < 0.05 was considered statistically significant.

## 3. Results

### 3.1. Curcumin Ameliorates PA-Induced HUVEC Injury

**Figure 1:**
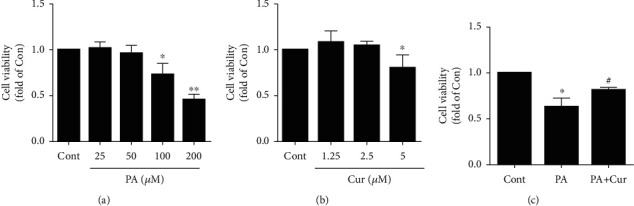
Effect of curcumin on HUVEC viability after PA treatment. (a). Dose-dependent effect of PA (25 *μ*M, 50 *μ*M, 100 *μ*M, and 200 *μ*M) on HUVEC viability after 24 h (*n* = 3; 1-way ANOVA with Dunnett's test). (b). Effect of curcumin (1.25 *μ*M, 2.5 *μ*M, and 5 *μ*M) on HUVEC viability after 24 h (*n* = 3; 1-way ANOVA with Dunnett's test). (c). HUVECs were exposed to PA (100 *μ*M) with or without pretreatment (30 min) with curcumin (2.5 *μ*M) for 24 h, and cell viability was measured using the CCK-8 assay (*n* = 4; 1-way ANOVA with Duncan's posthoc test). The data are shown as means ± SD (^∗^*P* < 0.05, ^∗∗^*P* < 0.01 vs. the Cont group; ^#^*P* < 0.05 vs. the PA group).

First, we sought to determine the effects of PA on HUVECs. After treatment with different concentrations (25, 50, 100, and 200 *μ*M) of PA for 24 h, cell viability was measured by CCK8. Cell viability was decreased in a dose-dependent manner after PA treatment (Figure 1(a)), while curcumin had no toxic effect on HUVECs less than 5 *μ*M (Figure 1(b)). Next, we treated HUVECs with 100 *μ*M PA with or without pretreatment with 2.5 *μ*M curcumin for 24 h, and PA treatment significantly reduced cell viability to 62% compared with control treatment (*P* < 0.05), while curcumin restored cell viability to 81% (Figure 1(c)).

**Figure 2:**
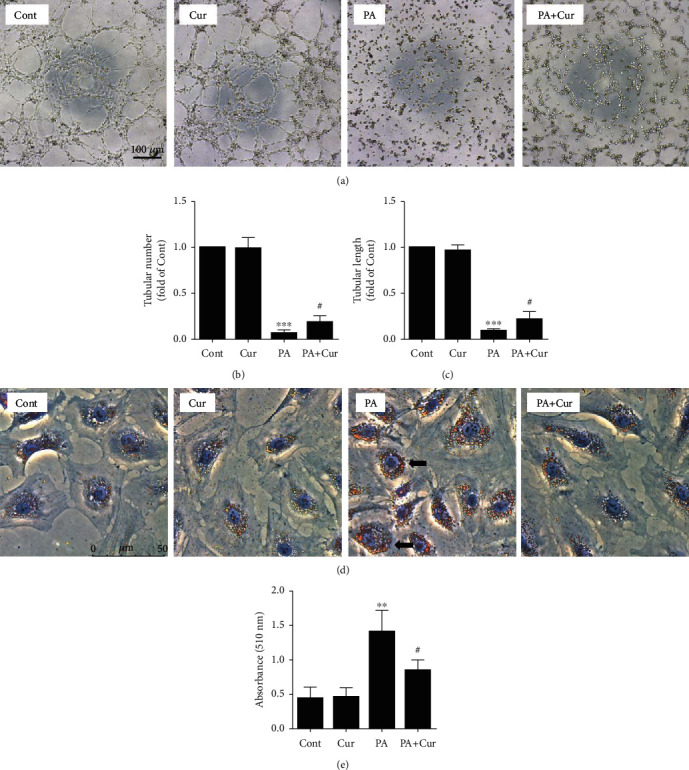
Effect of curcumin on HUVEC function and lipid accumulation after PA treatment.(a) Representative phase-contrast microscopy images of the HUVEC tube formation. Scale bar, 100 *μ*m. (a, b) Tube number and tube length were quantified using Image-Pro Plus software (*n* = 9; 1-way ANOVA with Bonferroni posthoc test). (d) Cells were stained with oil red O, and lipid accumulation was visualized under a microscope. Scale bar, 50 *μ*m. (e) The amount of cellular triglycerides was further quantified by measuring the absorbance at 500 nm using a thermomicroplate reader (*n* = 4; 1-way ANOVA with Duncan's posthoc test). The data are shown as means ± SD (^∗∗∗^*P* < 0.001, ^∗∗^*P* < 0.01 vs. the Cont group; ^#^*P* < 0.05 vs. the PA group).

Next, the tube formation assay was performed to evaluate the angiogenesis capacity of HUVECs. Under normal conditions, HUVECs formed tubules, while PA strongly impaired the angiogenesis ability of HUVECs. However, curcumin reversed this effect, as shown by more tubes and longer tube lengths (Figures 2(a)–2(c)). Additionally, Oil Red O staining was performed to assess lipid droplets. Massive lipid droplets were observed in the PA group, but the addition of curcumin effectively decreased lipid aggregation in HUVECs (Figure 2(d)). Furthermore, the lipid content was further quantified by isopropanol dissolution (Figure 2(e)). In summary, curcumin ameliorated PA-induced HUVEC injury.

### 3.2. PA Induces the LOX-1 Expression and ER Stress in HUVECs

**Figure 3:**
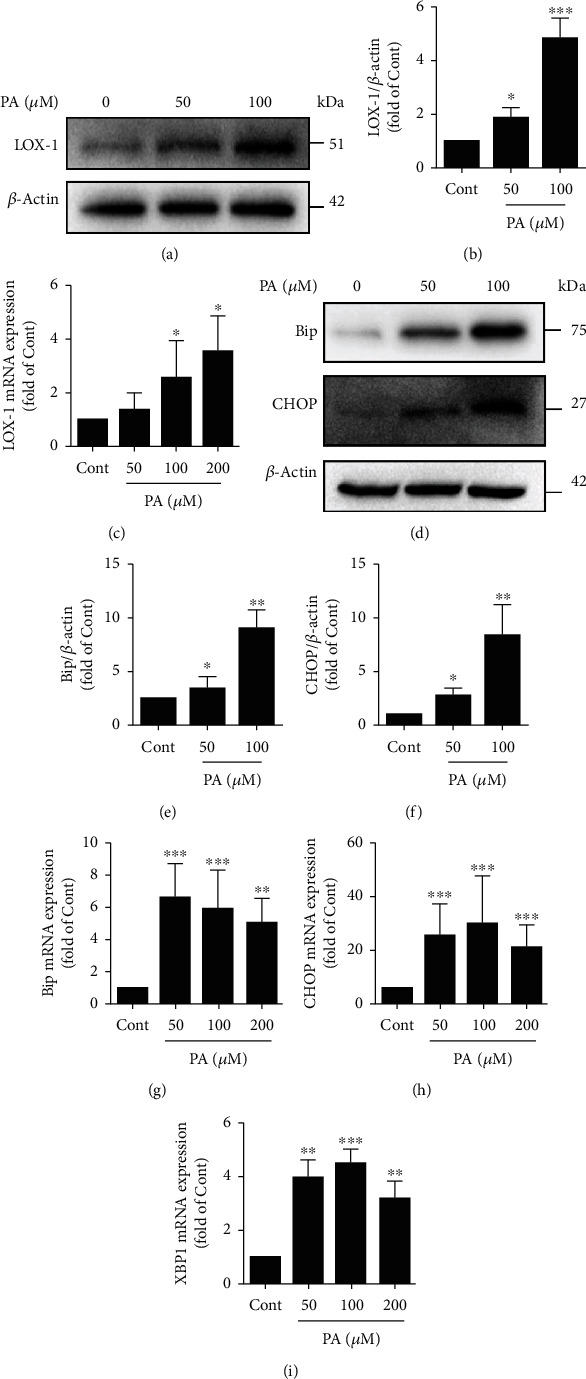
Effects of PA on LOX-1 and ER stress marker expression in HUVECs. (a) The protein expression level of LOX-1 was measured by Western blotting. (b) The expression of LOX-1 was quantified via densitometry using ImageJ software (*n* = 3; 1-way ANOVA with Duncan's posthoc test). (c) The mRNA expression level of LOX-1 was measured via quantitative PCR (*n* = 3; 1-way ANOVA with Duncan's posthoc test). (d) The protein expression levels of Bip and CHOP were measured by Western blotting. The expression of Bip (e) and CHOP (f) was quantified via densitometry using ImageJ software (*n* = 3; 1-way ANOVA with Duncan's posthoc test). (g–i) The mRNA expression levels of ER stress-related genes (*Bip, CHOP*, and *XBP1s*) were measured using quantitative PCR (*n* = 3; 1-way ANOVA with Duncan's posthoc test). The data are shown as means ± SD (^∗∗∗^*P* < 0.001, ^∗∗^*P* < 0.01, ^∗^*P* < 0.05 vs. the Cont group).

In our initial experiments, we sought to identify experimental conditions that cause LOX-1 upregulation in HUVECs. HUVECs expressed minimal levels of LOX-1 under normal conditions, but PA (50 and 100 *μ*M) induced LOX-1 protein upregulation (Figures 3(a) and 3(b)). Additionally, we examined the LOX-1 mRNA expression by qPCR and observed a dose-dependent increase in the LOX-1 gene expression after PA treatment (Figure 3(c)). In our previous study, we showed that ER stress is essential for PA-induced lipotoxicity in HUVECs. In this study, PA also obviously increased the Bip and CHOP expression in a dose-dependent manner (Figures 3(d)–3(f)). Furthermore, similar results were observed in quantitative PCR experiments: PA robustly upregulated the *Bip*, *CHOP*, and *XBP1s* mRNA levels in a dose-dependent manner after 24 h (Figures 3(g)–3(i)).

### 3.3. ER Stress Is Involved in PA-Induced LOX-1 Expression

**Figure 4:**
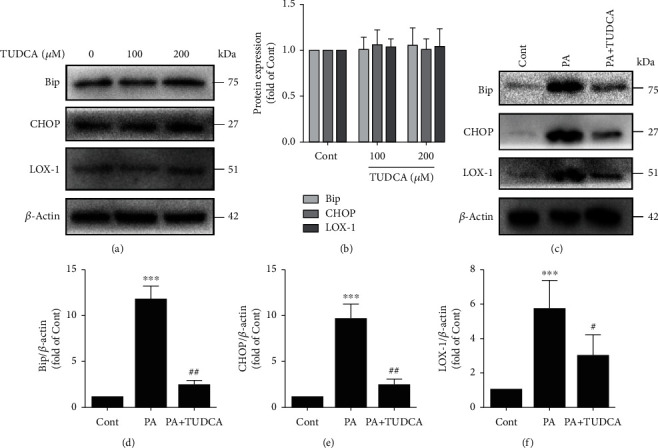
ER stress is involved in PA-induced LOX-1 upregulation. (a) The protein expression levels of Bip, CHOP, and LOX-1 were measured by Western blotting. (b) The expression levels of Bip, CHOP, and LOX-1 were quantified via densitometry using ImageJ software (*n* = 3; 1-way ANOVA with Duncan's posthoc test). (c) The protein expression levels of Bip, CHOP, and LOX-1 were measured by Western blotting. The expression levels of Bip (d), CHOP (e), and LOX-1 (f) were quantified via densitometry using ImageJ software (*n* = 4; 1-way ANOVA with Duncan's posthoc test). The data are shown as means ± SD (^∗∗∗^*P* < 0.001, ^∗∗^*P* < 0.01 vs. the Cont group;^##^*P* < 0.01, ^#^*P* < 0.05 vs. the PA group).

To determine whether PA-induced LOX-1 upregulation contributes to ER stress, we inhibited ER stress with TUDCA, a chemical ER stress inhibitor. TUDCA alone did not influence Bip, CHOP, or LOX-1 protein expression (Figures 4(a) and 4(b)) but significantly suppressed ER stress caused by PA in HUVECs (Figures 4(c)–4(e)). Notably, TUDCA also obviously decreased the PA-induced LOX-1 expression (Figures 4(c) and 4(f)), suggesting that alleviating ER stress contributes to the PA-induced amelioration of the LOX-1 expression. In summary, these results demonstrated that LOX-1 upregulation caused by PA in HUVECs is attributable to ER stress.

### 3.4. Curcumin Inhibits the PA-Induced LOX-1 Expression

**Figure 5:**
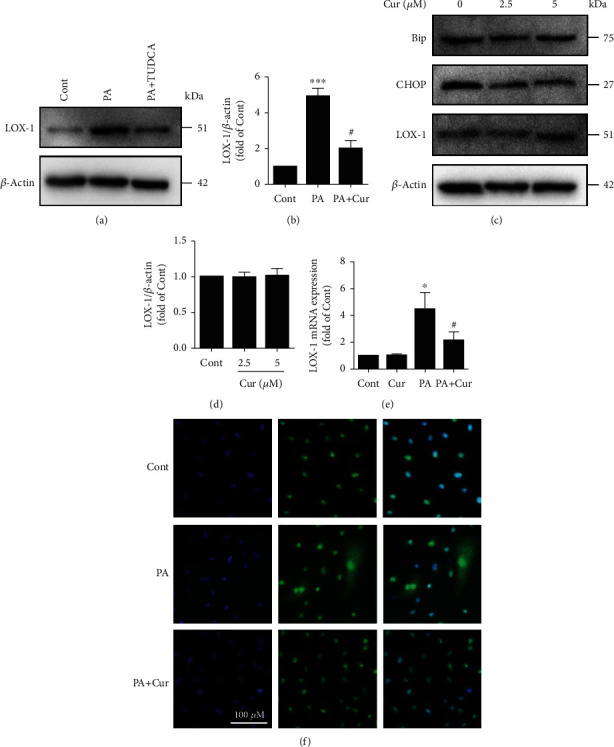
Curcumin inhibits PA-induced LOX-1 upregulation. (a) The protein expression level of LOX-1 was measured by Western blotting. (b) The expression was quantified via densitometry using ImageJ software (*n* = 3; 1-way ANOVA with Duncan's posthoc test). (c) The protein expression levels of Bip, CHOP, and LOX-1 were measured by Western blotting. The expression of LOX-1 (d) was quantified via densitometry using ImageJ software (*n* = 3; 1-way ANOVA with Duncan's posthoc test). (e) The mRNA expression level of LOX-1 was measured via quantitative PCR (*n* = 3; 1-way ANOVA with Duncan's posthoc test). (f) Images of immunofluorescence staining of LOX-1 in HUVECs in different groups. The data are shown as means ± SD (^∗∗∗^*P* < 0.001, ^∗^*P* < 0.05 vs. the Cont group;^#^*P* < 0.05 vs. the PA group).

Curcumin has protective effects against PA-induced lipotoxicity. However, the impact of curcumin on the LOX-1 expression under PA conditions in HUVECs that has not yet been reported. Consistent with our hypothesis, curcumin suppressed the protein expression of LOX-1 compared with that in the PA group (Figures 5(a) and 5(b)). Moreover, curcumin alone did not influence the LOX-1 protein level (Figures 5(c) and 5(d)). These results were also confirmed by quantitative PCR, which showed that curcumin distinctly decreased the LOX-1 gene expression compared with that in the PA group (Figure 5(e)). Furthermore, immunofluorescence staining showed that LOX-1 is increased in the cytoplasm after PA treatment, but this effect was reversed by the addition of curcumin (Figure 5(f)). Collectively, these results indicated that curcumin inhibits the LOX-1 expression induced by PA.

### 3.5. Curcumin Ameliorates ER Stress in HUVECs

**Figure 6:**
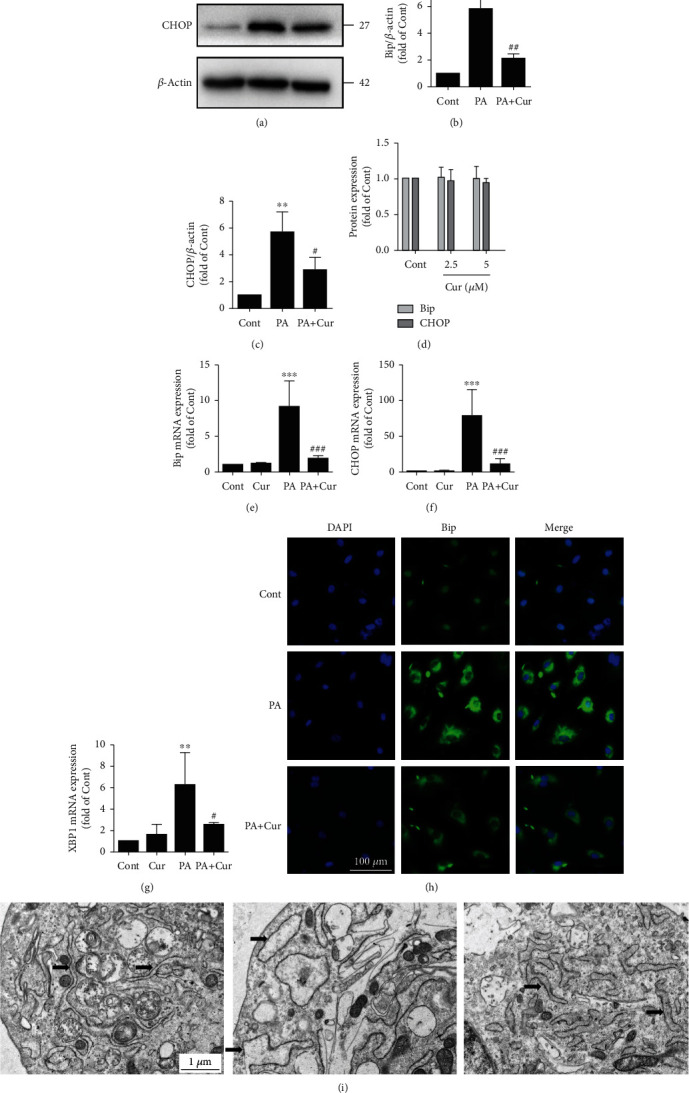
Curcumin ameliorates ER stress in HUVECs. (a) The protein expression levels of Bip and CHOP were measured by Western blotting. The expression of Bip (b) and CHOP (c) was quantified via densitometry using ImageJ software (*n* = 3; 1-way ANOVA with Duncan's posthoc test). (d) The expression of Bip and CHOP (Figure 5(c)) was quantified via densitometry using ImageJ software (*n* = 3; 1-way ANOVA with Duncan's posthoc test). (e–g) The mRNA expression levels of ER stress-related genes (*Bip*, *CHOP* and *XBP1s*) were measured by quantitative PCR (*n* = 4; 1-way ANOVA with Duncan's posthoc test). (h) Images of immunofluorescence staining of Bip in HUVECs in different groups. (i) Cellular ultrastructure in different groups. The arrows indicate endoplasmic reticulum. The data are shown as means ± SD (^∗∗∗^*P* < 0.001, ^∗∗^*P* < 0.01 vs. the Cont group;^###^*P* < 0.001, ^##^*P* < 0.01, ^#^*P* < 0.05 vs. the PA group).

Because ER stress contributes to LOX-1 upregulation caused by PA and curcumin suppresses the LOX-1 expression, we next investigated whether curcumin inhibits PA-induced ER stress in HUVECs. Consistent with our hypothesis, curcumin substantially suppressed the Bip and CHOP protein levels compared with PA (Figures 6(a)–6(c)), and curcumin alone did not affect the levels of Bip and CHOP under PA-free conditions (Figures 5(c) and 6(d)). Consistent with the Western blot results, curcumin also significantly decreased the expression of several ER stress-related genes, including *CHOP*, *XBP1s*, and *Bip* (Figures 6(e)–6(g)). Additionally, immunofluorescence staining was performed to detect the ER stress marker Bip, and the results indicated that curcumin reversed the elevated level of Bip caused by PA (Figure 6(h)). To obtain more detailed and informative evidence, we performed TEM analysis. Under normal culture conditions, the ER was regular and tubular. However, the ER became dramatically dilated after PA treatment, and this effect was visibly suppressed by curcumin (Figure 6(i)). Taken together, the above results indicated that curcumin profoundly alleviates ER stress induced by PA in HUVECs.

### 3.6. Curcumin Alleviates the PA-Induced LOX-1 Expression by Suppressing ER Stress

**Figure 7:**
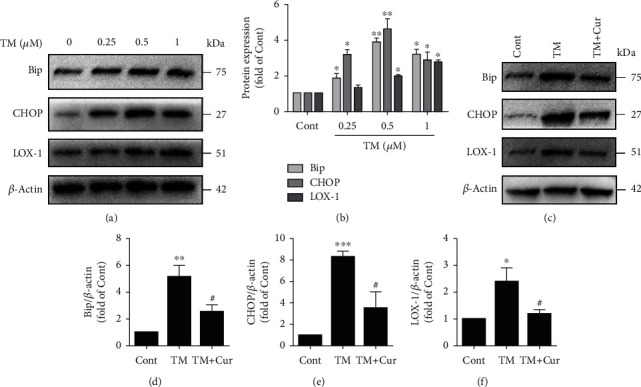
Curcumin alleviates PA-induced LOX-1 upregulation by suppressing ER stress. (a) The protein expression levels of Bip, CHOP, and LOX-1 were measured by Western blotting. (b) The expression levels of Bip, CHOP, and LOX-1 were quantified via densitometry using ImageJ software (*n* = 3; 1-way ANOVA with Duncan's posthoc test). (c) The protein expression levels of Bip, CHOP, and LOX-1 were measured by Western blotting. The expression levels of Bip (d), CHOP (e), and LOX-1 (f) were quantified via densitometry using ImageJ software (*n* = 3; 1-way ANOVA with Duncan's posthoc test). The data are shown as means ± SD (^∗∗∗^*P* < 0.001, ^∗∗^*P* < 0.01, ^∗^*P* < 0.05 vs. the Cont group; ^#^*P* < 0.05 vs. the PA group).

To determine whether curcumin inhibits the LOX-1 expression by suppressing ER stress, we introduced the ER stressor tunicamycin. Tunicamycin (TM) markedly induced the expression of LOX-1 and ER stress markers Bip and CHOP in HUVECs in a dose-dependent manner after 24 h of treatment (Figures 7(a) and 7(b)). Interestingly, curcumin completely blocked the tunicamycin-induced Bip and CHOP expression (Figure 7(c)–7(e)). Additionally, to determine whether the alleviation of ER stress by curcumin inhibits the LOX-1 expression, the LOX-1 protein levels were detected. As expected, curcumin inhibited the tunicamycin-induced expression of LOX-1 (Figures 7(c) and 7(f)). Thus, our current data suggest that curcumin alleviates the PA-induced LOX-1 expression by suppressing ER stress.

## 4. Discussion

SFAs exert harmful effects on many cell types, including pancreatic *β* cells, endothelial cells, and tumor cells. PA is one of the most common saturated fats and exists widely in plant oils and animal fats. After a long-term high-fat diet, more FFAs are released by the enlarged adipose tissue mass, and the elevated plasma FFAs inhibit the antilipolytic effect of insulin, which contributes to further FFA release into the circulation. FFAs can be taken up by endothelial cells and resolved into water and carbon dioxide in the mitochondria along with the release of large amounts of ATP. When excess FFAs are taken up by endothelial cells, some of them are stored in the form of triglycerides. Although triglyceride accumulation is considered beneficial against excess FFAs [25], endothelial cells may finally develop steatosis, which is called lipotoxicity [26]. In the present study, we treated HUVECs with PA (100 *μ*M) and observed reduced cell viability and angiogenesis ability, suggesting that cellular dysfunction and lipotoxicity occurred. Various studies have indicated that ER stress is crucial in PA-induced lipotoxicity [11]. Cao et al. found that PA induced ER stress, and that knockdown of the PERK gene expression significantly inhibited PA-induced apoptosis in liver cells, suggesting that ER stress is highly associated with lipotoxicity [27]. The ability of PA to increase ER stress in HUVECs was confirmed in this study by detecting increased Bip and CHOP levels. Furthermore, ultrastructural examination of HUVECs showed highly inflated ER in the PA group, suggesting that severe ER stress occurred.

Recent studies have shown that curcumin prevents hyperlipidemia-induced lipotoxicity in rats [28]. Furthermore, curcumin treatment inhibited PA-induced apoptosis, relieved mitochondrial depolarization, and increased the Bcl-2/Bax ratio in MIN6 pancreatic *β* cells [29]. Additionally, curcumin attenuates PA-induced apoptosis by suppressing ER stress in cardiomyocytes [24]. Consistent with these results, in our study, curcumin improved HUVEC viability and angiogenic capacity. Curcumin also significantly reduced lipid accumulation. Based on the above findings, we concluded that curcumin alleviates PA-induced lipotoxicity. However, the mechanisms involved in the protective effects of curcumin against PA-induced lipotoxicity must be further investigated. Kuo et al. showed that curcumin ameliorates SFA-induced mitochondrial dysfunction in hepatocytes [30]. Chen et al. reported that curcumin protects testicular Leydig cells against PA-induced apoptosis by modulating ER stress [31]. Similarly, curcumin decreased the PA-induced mRNA and protein expression of Bip and CHOP in this study. Hence, these results led to speculation that ER stress may be involved in curcumin-mediated protection against PA-induced injury in HUVECs. Further studies are needed to elucidate the contributions of the ER stress-associated signaling pathway to the effects of curcumin.

LOX-1 is the major receptor for oxLDL uptake by endothelial cells [32], and excessive uptake of oxLDL contributes to the initiation of AS [33]. Endothelial cells express minimal levels of LOX-1 under normal conditions, but LOX-1 is induced in vitro by proinflammatory, oxidative, and mechanical stimuli [5]. Hong et al. showed that Ox-LDL induces endothelial cell apoptosis via the LOX-1-dependent ER stress pathway [34]. Ishiyama et al. showed that PA induces LOX-1 upregulation along with increased uptake of oxLDL in macrophage cells [7]. Consistent with these results, PA upregulated the LOX-1 expression in HUVECs in our study. To gain insight into the mechanism underlying this effect, we evaluated the contribution of ER stress based on our previous study [3]. Interestingly, LOX-1 upregulation was alleviated by the ER stress inhibitor TUDCA, suggesting that suppressing ER stress lowers PA-induced LOX-1 expression. Additionally, the ER stress agonist tunicamycin also induced LOX-1 upregulation. Hence, we conclude that ER stress may contribute to PA-induced LOX-1 upregulation. However, ER stress is certainly not the only mechanism involved in the PA-induced LOX-1 expression. Cheng et al. reported that PA-induced hyperphosphorylation of Akt, ERK1/2, JNK1/2, and p38 MAPK is involved in LOX-1 upregulation in cultured aortic vascular smooth muscle cells [35]. Additionally, the LOX-1 expression is closely associated with oxidative stress [36]. Hence, the detailed molecular mechanisms of the PA-induced LOX-1 expression are multifaceted and must be further investigated.

Our subsequent study found that curcumin alleviates PA-induced LOX-1 upregulation. Several studies have shown that curcumin reduces the LOX-1 expression. Kang et al. showed that curcumin reduces Ang II-mediated upregulation of LOX-1 [37]. Lee et al. also found that curcumin inhibits the TNF*α*-induced LOX-1 expression [38]. Furthermore, curcumin suppresses the oxLDL-induced gene expression of LOX-1, and this suppressive effect is due to inhibition of the Wnt signaling pathway and activation of PPAR*γ* [39]. However, in this study, compared with TNF-*α* or oxLDL, PA mildly upregulated the LOX-1 mRNA and protein levels, suggesting that a different mechanism contributes to the PA-mediated increase in LOX-1. Hence, the key mechanism of curcumin-mediated inhibition of PA-induced LOX-1 upregulation may be different. In the present study, our results indicated that the suppression of ER stress by curcumin is crucial in this scenario. First, PA induced the LOX-1 expression along with elevated levels of ER stress, and alleviating ER stress by TUDCA disrupted the LOX-1 expression. Second, the elevated LOX-1 expression and ER stress was suppressed by curcumin. Curcumin completely blocked the upregulation of the Bip and CHOP expression induced by 100 *μ*M PA, suggesting that curcumin has powerful anti-ER stress effects. Third, curcumin also suppressed the tunicamycin-induced LOX-1 expression and Bip and CHOP upregulation. In conclusion, these results collectively indicate that curcumin alleviates the PA-induced LOX-1 expression mainly by inhibiting ER stress. The present study appears to be the first to show that curcumin attenuates the PA-induced LOX-1 expression by inhibiting ER stress in HUVECs. However, whether curcumin reduces the LOX-1 expression via its antioxidant and anti-inflammatory effects remains to be further explored. Additionally, whether curcumin has similar effects in vivo requires further experimental validation.

In conclusion, the present study indicates that curcumin mitigates PA-induced endothelial lipotoxicity and LOX-1 expression in HUVECs, and that ER stress plays a crucial role in these effects. The current study provides new insight into curcumin application to protect against endothelial injury and sheds light on the mechanism of the preventive effects of curcumin against atherosclerosis.

## Data Availability

The data used to support the findings of this study are available from the corresponding author upon request.

## Supplementary Materials

Supplementary Materials All of the primers used were listed in the supplementary material (supplementary Table S1).
