# Supplementary material for: Curcumin Alleviates Palmitic Acid-Induced LOX-1 Upregulation by Suppressing Endoplasmic Reticulum Stress in HUVECs
Source: Biomed Res Int. 2021 Aug 22;2021:9983725. doi: 10.1155/2021/9983725 (PMC8405307; doi:10.1155/2021/9983725)
Supplement: Supplementary Materials — All of the primers used were listed in the supplementary material (supplementary Table S1). [file 9983725.f1.docx]

| Gene | Forward | Reverse |
| --- | --- | --- |
| Bip | AAGAACCAGCTCACCTCCAA | CACCTTGAACGGCAAGAACT |
| CHOP | GAGAATGAAAGGAAAGTGGCAC | ATTCACCATTCGGTCAATCAGA |
| XBP1s | CCCTCCAGAACATCTCCCCAT | ACATGACTGGGTCCAAGTTGT |
| LOX-1 | CGACTCTAGGGGTCCTTTGC | GCTTCCGAGCAAGGGTTTCT |
|  |  |  |

**Table1: Primers used for q-PCR**
